# Cocaine self-administration attenuates brain glucose metabolism and functional connectivity in rats

**DOI:** 10.1371/journal.pone.0324522

**Published:** 2025-06-06

**Authors:** Christopher Rowan, Colin Hanna, Munawwar Sajjad, Rutao Yao, Alireza Sharafsha, Kai-Uwe Lewandrowski, Kenneth Blum, Albert Pinhasov, Panayotis K. Thanos

**Affiliations:** 1 Behavioral Neuropharmacology and Neuroimaging Laboratory on Addictions, Clinical Research Institute on Addictions, Department of Pharmacology and Toxicology, Jacobs School of Medicine and Biosciences, State University of New York at Buffalo, Buffalo, New York, United States of America; 2 Department of Nuclear Medicine, University at Buffalo, Buffalo, United States of America; 3 Cellular and Molecular Research Center, School of Medicine, Guilan University of Medical Sciences, Rasht, Iran,; 4 Department of Orthopaedics, Universidade Federal do Estado do Rio de Janeiro, Rio de Janeiro, Brazil; 5 Center for Sports, Exercise, and Mental Health, Western University Health Sciences, Pomona, California, United States of America; 6 Department of Molecular Biology, Adelson School of Medicine, Ariel University, Ariel, Israel; 7 Department of Psychology, Eotvos Loránd University, Institute of Psychology, Budapest, Hungary; SUNY Empire, UNITED STATES OF AMERICA

## Abstract

**Background:**

Cocaine abuse and Cocaine Use Disorder (CUD) is an increasingly urgent public health issue leading to major health risks often resulting in a decreased lifespan and quality of life. Previous human research has described brain function of cocaine addicts however the amount of cocaine use, duration of use, and exclusion of using other drugs (i.e., nicotine and alcohol) have all been difficult to control. One unanswered question is related to how does cocaine affect both brain glucose metabolism and functional connectivity?.

**Methods:**

The present study examined using positron emission tomography (PET) imaging and the glucose analog [^18^F]-Fluorodeoxyglucose (^18^F-FDG), brain glucose metabolism (BGluM) and functional connectivity in male rats (N = 6) that self-administered cocaine compared to baseline control scans in the same animals prior to cocaine exposure.

**Results:**

Our Results showed that Cocaine Self-Administration (CSA) caused significant BGluM decreases in several brain regions including posterior thalamic nuclei, Claustrum (Cl); Solitary nucleus, Presubiculum (PrS); Caudate Putamen (CPu); Anterior hypothalamic area (AHA); Ventral pallidum (VP); and amygdala. Activation (increased BGluM) was observed in the primary somatosensory cortex. These regions are associated with memory, spatial navigation, visual processing and saliency along with other somatosensory and motor functions, as well as regulatory autonomic function (cardiovascular) and hormonal response.

**Conclusion:**

This brain functional connectivity mapping illustrated a brain circuit composed of brain regions that are either a part of or connect with the mesolimbic reward pathway that is mediated by **dopamine**. When this circuit is dysregulated, it is believed to be associated with substance use disorders and reward dysregulation which have recently been described as attributes of preaddiction.

## 1. Introduction

The rate of reported cocaine uses and related deaths has increased in recent years along with the rate of Cocaine Use Disorder (CUD) [1]. Cocaine has detrimental effects on a user’s mental and physical health in both acute and chronic use. The physical effects include; increased blood pressure and risk for heart disease as well as profound damage to the respiratory and nervous systems [2–15]. Cocaine has many reported effects on the human brain including; damage to veins and arteries that may result in chronic headaches, stroke, seizures and/or seizure disorders [16–24].

Among the current literature there is supporting evidence for the inhibitory effect that cocaine exerts on brain glucose utilization in humans [25]. Most findings localize dysfunction mainly within cortico-striatal-thalamic circuitry and implicate reward systems in the pathology perpetuating persistent use and craving. Chronic cocaine use that is often associated with CUD is responsible for a plethora of cognitive deficits in humans including impairments in attention, memory, verbal fluency, sensory-perceptual functions, response inhibition, and impulsivity [26–29]. These findings are consistent with the reports on BGluM inhibition. However, specific schedule related and dose related effects of cocaine on the brain are difficult to capture. Preclinical models are useful for this purpose.

Preclinical cocaine data has shown regionally selective alterations in cerebral blood flow and glucose utilization following a single dose of cocaine and that this was associated with a marked decrease in matching task performance in rhesus macaque monkeys [30]. Additionally, cocaine was shown to increase dopaminergic signaling and motor activity in rats [31,32] which is believed to be mediated by a number of mechanisms including the blocking of dopaminergic reuptake via inhibition of dopamine transporter (DAT). Cocaine also induced adaptations in cortical regions known to relate to inhibitory avoidance such as cortical pyramidal neurons [33–35]. FDG PET imaging has been used effectively and extensively in animal models to measure the brain glucose response to drugs and therapeutic approaches [36–47]. Cocaine Self-Administration (CSA) is widely considered a gold standard approach for studying cocaine abuse behavior in rodents [48–50]. Prior studies in animals have found alterations in brain activity following CSA that are consistent with findings on glucose utilization in cocaine dependent subjects [51–53]. There are numerous studies linking cocaine use, and more specifically CSA, to dopamine mediated reward circuitry [54–57]. Cocaine abuse has been reported to lead to an imbalance of cortical and sub-cortical processes that produce a downstream effect leading to altered patterns of behavior reflected in decision-making, inhibitory control, and processing the salient features of social stimuli [58,59].

This collective body of research provides support and establishes the need for conducting the present study. Previously acquired clinical data on cocaine self-administration and brain glucose metabolism has produced significant findings pertaining to altered brain activity. However, this research having been done on humans, though significant, yields data that has limited reliability. The goal that was achieved with the present study is to examine the brain functional response to cocaine self-administration in a controlled animal model thus providing unique, clinically relevant, and novel preclinical data.

## 2. Methods

### 2.1. Animals

Male adolescent Sprague-Dawley rats (n = 6) were used for this within-subjects experiment. All rats were housed individually. Room temperature was held constant at 22.0 ◦C ± 2.0 ◦C. The lighting schedule was held on a 12-h reverse light/dark cycle (dark cycle: 8:00 a.m. to 8:00 p.m.). Rats were kept on an ad libitum diet. For the cocaine self-administration experiments, rat chow was restricted (18 g/day) to maintain a stable body weight, which was recorded daily throughout the experiment. All animals were handled daily to reduce the stress associated with handling. All experimental procedures were executed in compliance with the National Academy of Sciences Guide for the Care and Use of Laboratory Animals (1996). This experiment was approved by the University at Buffalo Institutional Animal Care and Use Committee.

### 2.2. Cocaine preparation

Cocaine hydrochloride (Sigma-Aldrich, St. Louis, MO) was prepared by dissolving it in 0.9% saline for doses of 0.750 mg/kg and 0.375 mg/kg for intravenous (i.v.) infusion at a volume of 100 μl. Most rat self administration studies use between.3 and 1 mg/kg and this is consistent with this convention [60]. Rats were anesthetized with isoflurane (2–3%) for surgery under aseptic conditions.

### 2.3. Apparatus

The self-administration apparatus (Habitest—Coulbourn Instruments; Allentown, PA) was placed inside a rigid foam sound attenuated cubicle equipped with a 28 V exhaust fan. Each operant chamber contained a horizontal grid floor with metal side walls and clear front and back walls. One side wall contained two levers and a food receptacle in the center. The left lever was designated as the active lever, whereas the right lever was the inactive lever. Both levers were situated directly under their respective cue lights. The back wall was equipped with an infrared activity monitor that collected locomotor behavior. Attached to a swivel arm, the infusion line entered the chamber from the center of the ceiling to be connected to the catheter on the rat for drug delivery. The cocaine was injected i.v. through the infusion line with an infusion pump at a fixed rate of 0.025 ml/s for duration of 4 s. All experimental variables were programmed and controlled using Graphic State Version 3.02 software that allowed for behavioral data collection.

### 2.4. Food training

**Fig 1:**
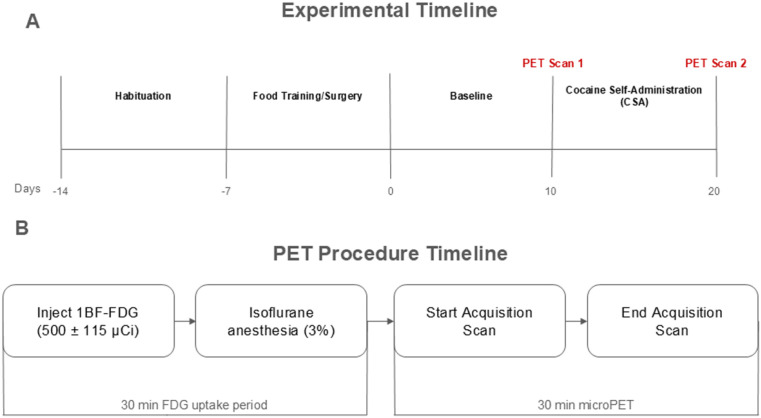
Experimental timeline: **(A)** Following a 7-day habituation period animals underwent food training then jugular vein catheterization (JVC) surgery. Animals were then kept at baseline for 10 days. Upon conclusion of the baseline period, each animal received a PET scan (“PET Scan 1” labeled in red). The following week, animals underwent cocaine self-administration (CSA) for ten days. Upon completion of CSA, each animal received another PET scan (“PET Scan 2” labeled in red). **(B)** Timeline of PET procedure: animals received [18F]-Fluorodeoxyglucose (FDG) via intraperitoneal injection. They were returned to their home cages for a 30-min uptake period. At the end of the uptake period, animals were anesthetized and placed in the bed of the PET R4 tomograph machine. PET scans lasted 30 min. After the scan, animals were recovered and returned to their home cages of the PET R4 tomograph machine. PET scans lasted 30 min. After the scan, animals were recovered and returned to their home cages.

After a 7-day habituation period rats were trained to respond to an operant lever response task for a food pellet before catheterization (Fig 1). Training sessions were conducted in the dark cycle from 8:00 h to 15:00 h and lasted for 4 days in 90 min daily sessions. A fixed-ratio 1 (FR1) reinforcement schedule with a 30 s timeout period was used. Pressing the active lever once released one (45 mg) food pellet into the food receptacle as the cue light was illuminated for a 30 s timeout period. During the timeout period, food was not released but the response recorded. Pressing the inactive lever had no programmed consequence.

Successful lever discrimination was achieved when rats met previously described criterion of an active/inactive lever press ratio ≥2:1 [61]. When rats exhibited lever discrimination they underwent surgery for catheterization. After surgery and recovery, rats went through one additional session of food retraining to ensure conditioning met aforementioned criterion to be able to move onto the cocaine self-administration phase of the experiment.

### 2.5. Jugular vein catheterization

Rats underwent jugular vein catheterization (JVC) surgery in preparation for cocaine self-administration (CSA). Techniques were adopted from previous literature [60,62]. Briefly, rats were anesthetized using 2–3% isoflurane (Fig 1). Throughout the surgery, breathing and the general health of the rats were monitored. Once the rat was anesthetized and pedal reflexes were checked, the surgery site was properly sterilized. A 3 cm horizontal incision was made in the upper lateral portion of the rats’ chest. Absorbable sutures were placed to anchor the catheter to the vein. Blunt dissection was used to tunnel to the dorsal portion of rat, where the port was pulled through. Once finalized, both the ventral and dorsal incisions were sterilely closed with absorbable sutures. JVC surgeries were followed by three consecutive days of post-operative care. During the post-operative period (3–7 days depending on the animal’s recovery), rats received both Rimadyl (5 mg/kg) and Baytril (5 mg/kg) via subcutaneous injections once a day for a minimum of three days, along with topical neomycin application to the incisions. Body weights and diet were carefully monitored to ensure the health and safety of the animals. In addition, catheters were flushed twice daily with heparin (30 units/mL), baytril (22.7 mg/mL) and saline, to maintain cannula patency.

### 2.6. Cocaine Self-Administration (CSA)

CSA sessions (90 min/day) lasted for 15 days in the dark cycle from 8:00 h to 15:00 h (Fig 1). A FR1 schedule was used with a 30 s timeout period. Immediately before and after the session, catheters were injected with saline to prevent occlusion. At the start of every session, rats received one priming infusion of cocaine. A single press of the active lever resulted in an immediate delivery of cocaine (0.75 mg/kg/infusion, i.v.) and a 30 s timeout period. During the timeout period, the cue light above the active lever was illuminated and the drug was not available. Lever presses were recorded during the timeout period. Inactive lever pressing during the session did not have a programmed consequence, but presses were recorded. During the first 7 days of cocaine self-administration, rats received an i.v. dose of 0.75 mg/kg/infusion cocaine in a volume of 0.1 ml with a FR1 schedule. For the last 8 days, the i.v. dose was halved to 0.375 mg/kg/infusion of cocaine under the FR1 schedule to look at the sensitivity in the dose response rate. Average amounts of cocaine consumed is described in Table 1.

**Table 1:** Mean cocaine consumed (mg/kg) over 10 days.

Day	1	2	3	4	5	6	7	8	9	10
Mean (mg/kg)	0.252	0.213	0.234	0.21	0.261	0.234	0.18	0.18	0.195	0.204
SEM	0.068	0.059	0.099	0.093	0.079	0.091	0.077	0.067	0.052	0.055

### 2.7. FDG PET imaging

Following food training, all rats were kept at baseline (no cocaine) for ten days, after which baseline PET scans were completed (Fig 1). Following baseline scans, rats completed ten days of cocaine self-administration, after which another PET scan was completed for each rat. All PET scans were performed as previously described [37–48]. Food was restricted for 8 h prior to scans to normalize and control blood glucose levels. Rats were injected with 500 ± 115 µCi of 18F-FDG (intraperitoneal injection). The uptake period lasted 30 min. After uptake, rats were anesthetized with isoflurane (3%, maintained at 1% for scan duration). Rats were secured to the bed of the scanner. PET scans lasted 30 min (as per standard imaging protocol). Scans were conducted using a Concorde Focus 120 microPET (Concorde Microsystems, Inc). Rats were returned to their home cages and given food and water after scans were completed. Brain glucose metabolism (BGluM) was assessed twice per animal, at the end of each period (baseline and CSA) With [^18^F] FDG (Cardinal Health, Franklin sq, NY).

### 2.8. Imaging and statistical analysis

**Fig 2:**
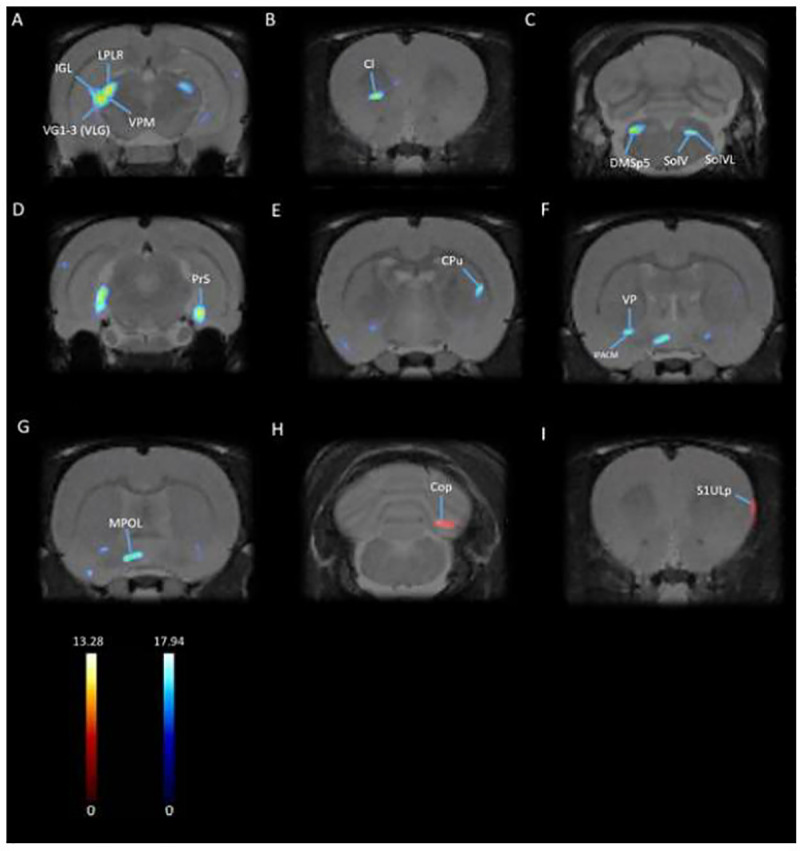
Significant clusters: Coronal PET images showing brain regions with significant (p < 0.001 and K > 50) metabolic inhibition (A-G) and activation (H-I) in rats following cocaine self-administration. T-values represent peak activation (t = 13.28) and inhibition (t = 17.94). Hot scale clusters illustrate BGluM activation in the **(H)** Cop and **(I)** S1ULp. Cold scale clusters represent inhibition, or decrease in BGluM, in **(A)** LPLR, IGL, VG, VPM, and LPMR; **(B)** Cl; **(C)** DMSp5, SolV, and SolVL; **(D)** PrS; **(E)** CPu; **(F)** VP and IPACM; **(G)** MPOL.

Completed PET scans were first reconstructed via MAP algorithm (15 iterations, 0.01 smoothing value, 256 × 256 × 256 resolution). Manual co-registration with an MRI template (63 slices) [63] was carried out in PMOD imaging software (http://www.pmod. com, RRID:SCR_016547, version 2.85). Low quality PET images were omitted. PET images are included in Fig 2. MatLab Software was used for automatic co-registration and spatial normalization (http://www.mathworks.com/products/matlab/, RRID:SCR_001622 R2018b). Statistical Parametric Mapping software (SPM8) was used to identify regional changes in BGluM. Significant metabolic differences between the experimental and control group were found using a paired t-test (N = 6) (significant voxel threshold K > 50, p < 0.001). Significant BGluM clusters were viewed in PMOD imaging software (version 2.85, PMOD Technologies). Activation clusters are colored in hot scale, while inhibition clusters are colored in cold scale. Mapping and labeling were carried out utilizing the rat brain atlas [63].

## 3. Results

Paired t-test showed that cocaine self-administration decreased BGluM (p < 0.001, K > 50; Fig 2, Table 2) compared to baseline in the following regions: Lateral posterior thalamic nucleus, laterorostral part (LPLR); mediorostral part (LPMR); Intergeniculate leaflet (IGL); Ventral geniculate nucleus (VG); Ventral posteromedial thalamic nucleus (VPM); Claustrum (Cl); Dorsomedial spinal trigeminal nucleus (DMSp5); Solitary nucleus, ventral part (SolV); Nucleus of the solitary tract, ventrolateral part (SolVL); Presubiculum (PrS); Caudate Putamen (CPu); Interstitial nucleus of the posterior limb of the anterior commissure, medial part (IPACM); Ventral pallidum (VP); Medial preoptic nucleus, lateral part (MPOL).

**Table 2:** Brain regions with significant metabolic inhibition (p < 0.001, K = 50) (A) and activation (B) in rats following cocaine self-administration compared to baseline. Cluster location is both noted and indicated by coordinates in stereotaxic space (medial-lateral, anterior-posterior, and dorsal-ventral). The t-value and Z-scores were calculated from the average BGluM of all voxels within the significant clusters. KE represents the number of voxels in the respective clusters. Each cell under “Brain Region” represents a separate cluster.

Brain Region	Location	Medial-Lateral	Dorsal-Ventral	Anterior-Posterior	t-Value	z-Score	KE
A							
LPLR LPMR IGL VG VPM	Thalamus	−2.8	4.6	−4.6	17.94	4.42	1141
Cl	Cortex	−2.0	4.6	3.2	14.71	4.20	346
DMSp5	Medulla	−2.8	7.8	−12.6	14.53	4.19	221
PrS	Hippocampus	3.8	7.2	−6.6	14.25	4.17	418
LPLC	Thalamus	2.6	4.6	−4.6	13.81	4.13	291
CPu	Basal Ganglia	4.4	5.4	−1.8	13.57	4.11	202
IPACM VP	Basal Ganglia	−3.2	8.0	−1.0	12.97	4.06	172
SolV SolVL	Medulla	1.6	8.0	−12.6	12.81	4.05	81
MPOL	Hypothalamus	−1.4	8.4	−0.6	12.68	4.04	147
B							
Cop	Cerebellum	2.6	5.8	−14.0	13.28	4.09	95
S1ULp.	Primary Somatosensory Cortex	5.4	4.6	2.2	11.18	3.89	142

BL < Cocaine (Activation).

BL > Cocaine (Inhibition).

Paired t-test also showed increased BGluM (activation) (p < 0.001, K > 50; Fig 2, Table 2) in the Copula of the pyramis (Cop) and the Primary somatosensory cortex, upper lip region (S1ULp) in PET scans following CSA compared to baseline PET scans. Complete details about cluster location, statistical significance, and voxel size can be seen in Table 2.

**Fig 3:**
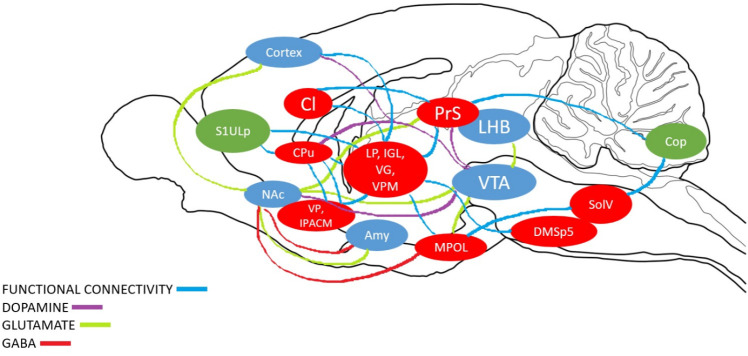
Sagittal drawing of hypothesized functional brain circuitry in response to cocaine self-administration. Activated/increased BGluM clusters are shown in green. Inhibition of BGluM is shown in red. Purple boxes represent brain regions which act as relay points for our clusters as well as regions involved in mesolimbic reward circuitry (Cortex, NAc [Nucleus Accumbens], Amy [Amygdala], VTA [Ventral Tegmental Area], LHb [Lateral Habenula]. Blue arrows indicate functional connectivity between significant regions discovered in our study. Purple arrows represent dopamine neurotransmission in the mesolimbic pathway. Green (Glutamate) and Red (GABA) dotted arrows represent excitatory and inhibitory neurotransmission in mesolimbic reward pathway respectively.

Cluster locations and regions were used for brain mapping and represented as the functional connectivity circuit in response to cocaine self-administration (Fig 3). Also indicated in our results is a decrease in functional connectivity in all regions of interest excluding the Cop and S1ULp. This was determined based on regional changes in brain glucose metabolism, which is a known indicator for altered functional connectivity. Circuitry with altered functional connectivity can be seen in Fig 3 indicated in blue, with distinct decreases in functional connectivity noted in pathways between regions colored in red.

## 4. Discussion

### 4.1. Thalamic inhibition

The thalamus is a crucial brain region when it comes to behavioral regulation and is widely regarded as a relay between sensory information (excluding olfaction) and cortical processing [64]. Though destination depends largely on the role of the thalamic subregion, most thalamocortical interaction involves thalamic projections to the cortex carrying sensory information [65]; however, some areas of the cortex do send efferent projections to the thalamus [66], thus establishing bidirectional interaction. With regards to cocaine use, the thalamus is broadly implicated in cocaine addiction pathology [67]. Thalamic inhibition is cited as a common biological marker for cocaine addiction and is believed to accompany an impairment in response inhibition [68]. One of the most notable findings in the present study is inhibited glucose metabolism within cortico-striatal-thalamic circuitry potentially resulting from the vasoconstricting effects of cocaine or the inhibition of neurotransmitter transporters (DAT, SERT, NET) [69,70]. This circuit has widespread functioning with regards to behavior, specifically pertaining to executive function [71]. Furthermore, parts of the thalamus have been identified to moderate glutamate levels in the striatum [72]. Inhibition of the thalamus can very easily disrupt this circuitry which would likely lead to executive control dysfunction, failed response inhibition, and cocaine seeking behavior as witnessed in our CSA paradigm. It is important to note that multiple studies have identified decreased neurotransmitter functioning, epscifically GABA and glutamate, have been linked to psychotic symptoms such as disorganization [73,74].

In addition to the aforementioned proposed roles of the thalamus, its subregions have more distinct and fine-tuned roles and functional connectivity. Specifically, the lateral posterior thalamic nucleus (LP), along with its subregions where inhibited BGluM was observed as well as adjacent regions are believed to have a role in visual attention and salience response [75,76]. As described, altered salience attribution and response inhibition are recognizable markers of addiction. Other subregions of the thalamus that showed decreased BGluM in this study are the ventral posteromedial thalamic nucleus (VPM) which is involved in sensory relay to the primary somatosensory cortex [77], as well as the ventral geniculate nucleus (VG) and the intergeniculate leaflet (IGL) which both have been related to mechanisms involving cocaine relapse [78].

### 4.2. Striatal inhibition

Striatal functioning, as mentioned above, is believed to be a very important aspect of substance use disorders. This is largely due to its role in reward circuitry. Striatal inhibition can clearly cause dysfunction of this circuit, resulting in the aforementioned impairment in executive control and response inhibition. Information from the striatum is also sent to structures of the pallidum and subsequently to the ventral tegmental area [79–81]. The ventral tegmental area is then responsible for the output of dopamine which determines the level of reward and reinforcement experienced in a given behavior [82]. It has been shown that often times the reward cue, encoded by the amygdala, can become conflated with the reward, even if it is not a desirable reward. This is generally believed to be based on the salience of the cue and may be dependent on internal physiological states, such as depletion of salt (with salt water as an undesirable reward re encoded to be desirable after depleted state), and phasic and tonic states of dopamine in the striatum [83]. Recent research has shown that brief phasic dopamine release in the nucleus accumbens and dorsolateral striatum reflects not only the size of the reward but also the effort already invested, or sunk cost. Interestingly, when animals were more motivated, their phasic dopamine response to reward was reduced. Even artificially triggered stimulation was shaped by sunk cost. These findings suggest that dopamine in the striatum simultaneously tracks cost, benefit, and motivation, though each on different timescales [84].

The dopamine depleting effects of cocaine dependency act on the striatum more than any other brain region [40,85–89]. This reduction in dopamine could be sufficient to reduce brain glucose metabolism in the region [90,91]. Reduced striatal activity can signify a reduction in direct striatal dopaminergic function and indirect striatal cortical dopaminergic function, which can lead to decreased reward states and increased drug-seeking behavior [92]. This is not always the case, however. There are some studies noting an increase in certain functions of the striatum such as functional connectivity as a result of CSA [93].

FDG PET has proven to produce reliable results regarding altered functional connectivity and this has been previously demonstrated by our lab and others [68,94–101]. Interestingly, increased functional connectivity in the striatum has been linked to improved smoking cessation [102] indicating a potential for compensatory brain activity following damage, which in this case would be CSA [103]. Additionally, parts of the striatum have been linked to a role in reward and decision making having a function in rational and irrational behavior [104,105].

### 4.3. Parahippocampal inhibition

Parahippocampal subregions of the hippocampus in the rodent brain include the perirhinal, postrhinal, and entorhinal cortices, as well as parts of the subicular complex. The parahippocampal cortex and the subicular complex are believed to be involved in cognition, memory/memory retrieval, and limbic activity [106,107]. Hanna et al found that exercise attenuates brain glucose metabolism in the post/parasubiculum in response to acute cocaine [39]. Specifically, the presubiculum of the subicular complex (a region of interest in our study) is primarily believed to play part in the hippocampus’ head direction system [108]. However, further evidence suggests that particular subicular subregions are involved in memory and emotion systems that regulate cocaine-seeking behavior in CSA rats [109–111]. Notably, these behaviors are often witnessed alongside an increase in subicular activity, which was not observed in the present study. Despite this, it is possible that any disruption in circuit activity could be related to the proliferation of addictive behavior; especially considering the apparent link between presubicular atrophy and impaired episodic memory and self-overconfidence in schizophrenic patients [112]. It is possible that these areas of the brain have a large part in the memory aspect of cocaine addiction, more specifically as it relates to cocaine-seeking behavior and that the observed decrease in brain glucose utilization in these regions is a result of altered neurotransmitter signaling from other regions.

There is also a significant cognitive component in the pathology of substance use disorders. Interestingly, some research has linked the presubiculum and the subiculum to cognitive impairment in Alzheimer’s Disease (AD), believing a loss in volume of these regions to be the first anatomical marker of AD [113,114]. While not directly addressing addiction pathology, this insight offers pertinent clues into the potential role of parahippocampal regions in reward and addiction.

### 4.4. Claustrum inhibition

The claustrum is a subcortical region situated between the insula and the putamen in both hemispheres. Proposed function of the claustrum includes strong involvement in higher order processes due to its widespread cortical connections [115,116]. It is believed that the claustrum sends inhibitory input to the cortex, specifically to the prefrontal cortex [117]. It is well supported that dysregulation of the prefrontal cortex is largely associated with addictive behavior and an inability to control reward seeking [118]. The claustrum has also become the subject of numerous studies involving attention, salience processing, and consciousness [117,119–122], any of which could prove to have a strong impact on the development of addiction. Salience processing and the misattribution of salience, for example, is proposed to be responsible for the hypersensitization to drugs and drug cues [58,123–125].

### 4.5. Ventral pallidum inhibition

The ventral pallidum is a part of the basal ganglia and has functional significance with regards to the reward pathway. Evidence supports the theory that cocaine self-administration results in a lack of synaptic transmission in the ventral pallidum that is consistent with our findings of inhibition of glucose utilization in the ventral pallidum [126]. This effect is thought to be an indirect result of cocaine’s action on the nucleus accumbens which has strong input to the ventral pallidum [57,127,128]. There is also the belief that this inhibitory response is serotonin mediated concluded in studies using serotonin receptor knockout mice and serotonin transporter (SERT) blockers [129]. This is believed to be due to the effects of cocaine on serotonin levels in the raphe nuclei and its projections to the ventral pallidum. Regarding reward circuitry, the ventral pallidum has a large role in encoding and sending reward related information to the ventral tegmental area [130,131], showing strong participation in the development of addiction and other reward related dysfunctions through the integration of stimulus information.

### 4.6. Hypothalamic inhibition

Chronic cocaine use is noted in the literature to lead to hypothalamic dysfunction as well as the related diminishing of natural rewards [132]. For example, individuals with cocaine use disorder are more likely to experience activation of the hypothalamus in response to cocaine cues when compared to natural rewards like food [133]. This does not refute our findings, as the observed decrease in hypothalamic glucose utilization occurred in the absence of cocaine cues.

### 4.7. Somatosensory activation

It may be perplexing that cortical activation is related to cocaine addiction, especially given the information presented above on cortical dysfunction in cocaine use. However, the present findings indicate increases in specific regions of the primary somatosensory cortex. S1 provides an understanding of the environment via sensory receptors in the periphery [134]. Notably, whiskers are a very important vessel for sensory input to the rat. This could help to explain the increase in glucose utilization to particularly the upper lip region of S1 (where whiskers are present or nearby) in this study [135]. Interestingly, altering of activity in somatosensory regions is believed to be a common result of cocaine exposure as well as having a role in the proliferation of craving and drug seeking behavior [136,137]. This is potentially a result of somatosensory responses to cocaine cues. With this information in mind, it is not difficult to deduce that activation of the somatosensory regions observed in this study is both in alignment with present beliefs and involved in the cyclical reinforcement of addiction over time.

### 4.8. Cerebellar activation

Activation of the Copula of the Pyramis in the cerebellum (as well as S1) has been observed in a study previously done by our team detecting exercise as a potential remedy for the adverse effects of cocaine on the brain [40] as well as another study noting increased blood flow to the copula as a result of treadmill walking [138].

### 4.9. CSA effects on dopamine and reward circuitry

Cocaine self-administration is a reliable model for cocaine use disorder (CUD). As such, it is known to have maladaptive effects on the brain’s dopamine reward circuitry [54]. Specifically, cocaine use has been shown to have vast effects on mesolimbic neuroplasticity via chemically altered signaling cascades, growth factors, etc. [139] as well as circuit wide alterations in dopamine receptor type and density [140]. A study conducted [141] concluded that D2R receptor density in the nucleus accumbens and CSA are negatively correlated indicating a potential inhibitory reward-seeking function for D2R receptors in reward circuitry. Dopamine D1 and D2 receptor activity are associated with altered glucose utilization in the rat basal ganglia [91]. A potential conclusion based on the given evidence is that CSA chemically and mechanically alters dopamine transmission to cause dysfunction of the reward pathway and thus altered reward seeking behavior and diminished response inhibition via dysregulated excitatory/inhibitory neurotransmission. Significant regions in the present study, as mentioned, show altered excitatory and inhibitory input via glucose utilization. Therefore, an invigorated cocaine-seeking behavioral output in our CSA paradigm can be mechanistically tied to altered dopaminergic reward signaling.

### 4.10. Genetic role of dopaminergic dysregulation with cocaine

Noble et al. [88] determined the relationship between specified allelic prevalence of the D2 dopamine receptor (DRD2) gene and family history/behavioral measures to have a positive and significant correlation. The prevalence of the A1 allele in cocaine dependent (CD) Caucasian (non-Hispanic) subjects (50.9%, n = 53) was significantly higher than either the 16.0% prevalence (P < 10(−4)) in non-substance abusing controls (n = 100) or the 30.9% prevalence (P < 10(−2)) in population controls (n = 265). Similarly, the prevalence of the B1 allele in CD subjects (38.5%, n = 52) was significantly higher than non-substance abusing controls (13.2%, n = 53). Logistic regression analysis of CD subjects identified (1) potent routes of cocaine use, (2) the interaction of early deviant behaviors, and (3) parental alcoholism as significant risk factors positively correlated with A1 allelic prevalence. This data suggests that a gene located on the q22-q23 region of chromosome 11 is related to cocaine dependence susceptibility.

Moreover, Huggett et al., [142] investigated the genetic and molecular architecture of cocaine dependence (CD) and cocaine use by integrating genome-/transcriptome-wide analyses In a 3176 cohort (74% having CD) detected a significant single-nucleotide polymorphism heritability of 28% for CD and identified three genes (two loci) underlying this predisposition: the C1qL2 (complement component C1 q like 2), KCTD20 (potassium channel tetramerization domain containing 20), and STK38 (serine/threonine kinase 38) genes. The same group also showed differentially expressed genes/transcripts in humans were enriched for the genes nominally associated with CD via GWAS (P < 0.05) and for differentially expressed genes in the hippocampus of cocaine-exposed mice [142]. Interestingly, their findings related to KCTD20 as a central component of a hippocampal gene network strongly associated with human cocaine use, agrees with others [143].

### 4.11. In deep silico PGX analyses related to the interaction of cocaine, glucose metabolism and functional connectivity

Our team evaluated via PGX the interaction of cocaine, glucose metabolism and functional connectivity utilizing in deep silico PGX analyses [144]. This work utilized PharmGKB to extract the PGx annotations related to Cocaine, Glucose, and Dopamine (Raw data). We performed filtering steps, refined, unrepeated, and brain-expressed genes combined in a list (49 genes) and checked in a Protein-Protein Interaction (PPI) network by STRING-MODEL to identify the top candidate genes [145]. Moreover, targeting potential protein-coding genes (having the most connections), *COMT, DRD2,* and *SLC6A3* and their connections were found (17 genes). Gene-miRNA Interactions (GMIs) by NetworkAnalyst revealed that *COMT*, *DRD2* and hsa-miR-16-5p have multiple interactions with *OPRM1,* and *BDNF*. Enrichr applied for Enrichment identified that the refined list of 17genes impact dopamine function and are interactive with dopaminergic pathways. Additionally, Substance Use disorders (SUD) was the most significant manifestation predicted for the interactiveness among these genes [146]. We are proposing herein that these PGx-based in silico analysis provided reliable strong validated connections based on the prior published data and highly accurate computational predictions. Notably, the *COMT gene was found be* the best-scored gene in our analyses [147].

## 5. Conclusion

This paper helps to provide further insight into the effects that cocaine self-administration has on glucose metabolism in the brain. Widely encompassing and clinically relevant circuitry and function including reward circuitry, salience attribution, behavioral output, and critical relay as well as information integrating regions such as the thalamus, ventral pallidum, and claustrum were discovered to be of great importance. Many brain regions implicated in this study have been known to feed into reward circuitry via dopaminergic communication. This research provides groundwork for further studies into CSA and brain glucose utilization potentially pertaining to human and/or female models to discover mechanisms driving behavior. Future studies may aim to conduct similar research incorporating different biological and/or environmental variables with a focus on uncovering mechanisms that may link altered regional brain glucose metabolism to behavioral changes to further refine understanding and efficacy of therapeutic intervention.

### 5.1. Limitations

Limitations to this study include it being limited to male rats. Female exclusive experiments as well as sex comparisons have been done and will be done in the future. Additionally, systemic metabolic changes may have contributed to the significant metabolic changes post cocaine administration.
