# Correction: Cocaine self-administration attenuates brain glucose metabolism and functional connectivity in rats

**DOI:** 10.1371/journal.pone.0332508

**Published:** 2025-09-15

**Authors:** Christopher Rowan, Colin Hanna, Munawwar Sajjad, Rutao Yao, Alireza Sharafshah, Kai-Uwe Lewandrowski, Kenneth Blum, Albert Pinhasov, Panayotis K. Thanos

The fifth author’s name is spelled incorrectly. The correct name is: Alireza Sharafshah. The correct citation is: Rowan C, Hanna C, Sajjad M, Yao R, Sharafshah A, Lewandrowski K-U, et al. (2025) Cocaine self-administration attenuates brain glucose metabolism and functional connectivity in rats. PLoS One 20(6): e0324522. https://doi.org/10.1371/journal.pone.0324522
